# A Mitochondrial Specific Antioxidant Reverses Metabolic Dysfunction and Fatty Liver Induced by Maternal Cigarette Smoke in Mice

**DOI:** 10.3390/nu11071669

**Published:** 2019-07-21

**Authors:** Gerard Li, Yik Lung Chan, Suporn Sukjamnong, Ayad G. Anwer, Howard Vindin, Matthew Padula, Razia Zakarya, Jacob George, Brian G. Oliver, Sonia Saad, Hui Chen

**Affiliations:** 1 School of Life Sciences, Faculty of Science, University of Technology Sydney, Sydney, NSW 2007, Australia; 2 Respiratory Cellular and Molecular Biology, Woolcock Institute of Medical Research, The University of Sydney, Sydney, NSW 2037, Australia; 3 Department of Clinical Chemistry, Faculty of Allied Health Sciences, Chulalongkorn University, Pathum Wan, Bangkok 10330, Thailand; 4 Graduate School of Biomedical Engineering, University of New South Wales, Sydney, NSW 2052, Australia; 5 Storr Liver Centre, The Westmead Institute for Medical Research, Westmead Hospital and The University of Sydney, Sydney, NSW 2037, Australia; 6 Kolling Institute of Medical Research, Royal North Shore Hospital, The University of Sydney, Sydney, NSW 2065, Australia

**Keywords:** ROS, liver fibrosis, MitoQ, glucose intolerance

## Abstract

Maternal smoking leads to glucose and lipid metabolic disorders and hepatic damage in the offspring, potentially due to mitochondrial oxidative stress. Mitoquinone mesylate (MitoQ) is a mitochondrial targeted antioxidant with high bioavailability. This study aimed to examine the impact of maternal cigarette smoke exposure (SE) on offspring’s metabolic profile and hepatic damage, and whether maternal MitoQ supplementation during gestation can affect these changes. Female Balb/c mice (eight weeks) were either exposed to air or SE for six weeks prior to mating and throughout gestation and lactation. A subset of the SE dams were supplied with MitoQ in the drinking water (500 µmol/L) during gestation and lactation. Intraperitoneal glucose tolerance test was performed in the male offspring at 12 weeks and the livers and plasma were collected at 13 weeks. Maternal SE induced glucose intolerance, hepatic steatosis, mitochondrial oxidative stress and related damage in the adult offspring. Maternal MitoQ supplementation reduced hepatic mitochondrial oxidative stress and improved markers of mitophagy and mitochondrial biogenesis. This may restore hepatic mitochondrial health and was associated with an amelioration of glucose intolerance, hepatic steatosis and pathological changes induced by maternal SE. MitoQ supplementation may potentially prevent metabolic dysfunction and hepatic pathology induced by intrauterine SE.

## 1. Introduction

Tobacco cigarette smoking during pregnancy has been associated with an increased risk of metabolic disorders in the offspring, especially type 2 diabetes in adulthood [1]. In utero smoke exposure (SE) increases oxidative stress and damage in the offspring’s liver [2], which can lead to mitochondrial dysfunction. Mitochondria are essential for glucose and fatty acid metabolism to generate energy in the form of ATP. The process of mitochondrial oxidative phosphorylation results in the generation of reactive oxygen species (ROS), which are normally cleared by endogenous mitochondrial antioxidants [3]. Within the mitochondria, the imbalance between excessive ROS and antioxidant capacity leads to oxidative stress and mitochondrial damage. Adaptive mechanisms respond to damaged mitochondria through the processes of mitophagy and mitochondrial biogenesis [4]. However hepatic mitochondrial dysfunction (increased oxidative stress or impaired adaptive mechanisms) can lead to glucose intolerance and triglyceride accumulation, which could underlie the development of type 2 diabetes and non-alcoholic fatty liver disease [5]. Increasing evidence, including our own work, has suggested that in utero SE can induce mitochondrial damage in multiple organs, increasing mitochondrial ROS output and impairing metabolic function [6,7,8,9]. Therefore, the impacts of maternal SE on metabolic disorders in the offspring could be related to liver mitochondrial damage. As a result, the liver was chosen as the focus of this study because it represents an important and novel field of investigation.

Mitoquinone mesylate (MitoQ) is a mitochondrial targeted antioxidant with high oral bioavailability, derived from covalently attaching a triphenylphosphonium cation moiety to Coenzyme Q10 (CoQ10) [10]. CoQ10 is an endogenous antioxidant with reduced levels in the plasma of smokers [11] and diabetic patients [12]. The positively charged moiety of MitoQ promotes its accumulation within the negatively charged inner mitochondria membrane, directly reducing mitochondrial oxidative stress [10]. We have demonstrated that maternal MitoQ supplementation during gestation and lactation alleviates the adverse effects of maternal smoking in the offspring’s lung and kidney [13,14]. Additionally, in a phase II clinical trial on patients with chronic hepatitis C, oral MitoQ administration decreased plasma alanine aminotransferase (ALT) activity, a marker of liver damage [10]. Thus, in a mouse model, we aimed to determine if maternal MitoQ supplementation during gestation and lactation could ameliorate the metabolic dysfunction and hepatic damage caused by SE.

## 2. Materials and Methods

### 2.1. Animal Model

The animal experiments were approved by the Animal Care and Ethics Committee of the University of Technology Sydney (ACEC #2014-638 and ACEC #2016-419) and carried out according to the Australian National Health and Medical Research Council Guide for the Care and Use of Laboratory Animals. After acclimatisation, virgin female Balb/c mice (eight weeks old, Animal Resource Centre, WA, Australia) were randomly assigned to two groups with equal starting body weight. Mice were either exposed to ambient air (Sham group, *n* = 9) or cigarette smoke (SE group, *n* = 19) generated from two cigarettes (Winfield Red, ≤16 mg tar, ≤1.2 mg nicotine, and ≤15 mg of CO; VIC, Australia) twice daily for six weeks prior to mating and throughout gestation and lactation as previously described [15]. The female mice were exposed to the smoke generated by each cigarette for 15 minutes, with a 5-minute interval between the two cigarettes. As indicated by plasma cotinine concentrations in both mothers and offspring, this model represents light human smokers, as we have previously published [8,16]. At mating, a sub-group of the SE dams were administered with MitoQ in the drinking water (500 µmol/L) during gestation and lactation (SE + MQ group, *n* = 9) [13]. MitoQ in the drinking water provided to the dams has previously been shown to reach the neonatal liver [17]. Male breeders and suckling pups were not exposed to cigarette smoke.

**Figure 1:**
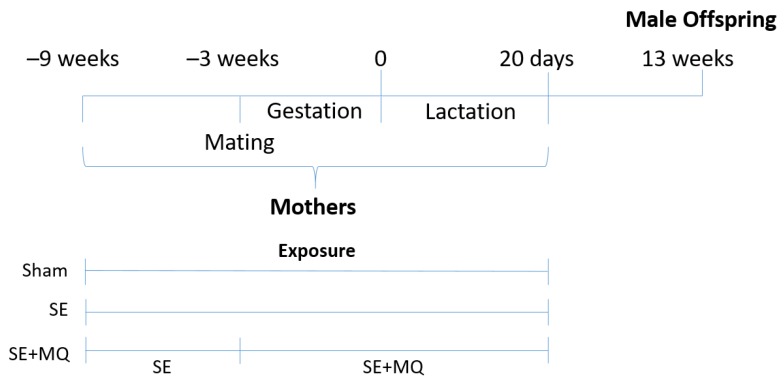
Schematic diagram of animal experiments. SE: cigarette smoke exposure, SE + MQ: SE with mitoquinone mesylate (MitoQ) supplementation.

Male offspring were studied because they are more susceptible to the adverse impacts of maternal smoking, as elucidated in our previous studies [8,15]. Male offspring were weaned at postnatal day 20 and maintained without additional intervention until 12 weeks of age, when an intraperitoneal glucose tolerance test (IPGTT) was performed as previously described [18]. After 5 hours of fasting, baseline blood glucose levels were measured followed by glucose injection (2 g/kg, ip). Blood glucose was measured at 15, 30, 60, and 90 minutes post-injection. The area under the curve (AUC) of the blood glucose curve was calculated for each mouse. At 13 weeks of age, after the induction of deep anaesthesia (4% isoflurane), blood was collected via cardiac puncture and the plasma was stored at −20 °C for further analysis. The livers were weighed and then snap frozen at –80 °C or fixed in 10% neutral buffered formalin for further analysis. One male offspring from each litter was used for tissue analyses (Figure 1).

### 2.2. Bioassays

Plasma ALT activity was measured using the Alanine Transaminase Colorimetric Activity Assay Kit (Cayman Chemical, Ann Arbor, MI, USA) according to the manufacturer’s instructions. Plasma insulin concentration was measured by ELISA (Abnova, Taiwan) according to the manufacturer’s instructions. Liver lipids were extracted using the Folch method [19]. Briefly, liver samples were homogenised in a chloroform:methanol (2:1) mixture. After agitation, the samples were washed with 0.6% saline solution and centrifuged at low speeds. The lower organic phase was then extracted and dried. Plasma, liver extracts and glycerol standards (Sigma-Aldrich, MO, USA) were incubated with triacylglycerol reagent (Roche Diagnostics, Basel, Switzerland) using an in-house assay [18]. 

### 2.3. Real Time-PCR

Total mRNA was extracted from frozen liver tissue with TriZol reagent (Life Technologies, Carlsbad, CA, USA) and first strand cDNA was generated using M-MLV Reverse Transcriptase, RNase H, Point Mutant Kit (Promega, Madison, WI, USA). Target gene expression was quantified with manufacturer pre-optimised and validated TaqMan primers and probes (Table S1, Thermo Fisher, San Jose, CA, USA) and standardised to 18S RNA. The probes of the target genes were labelled with FAM and those for housekeeping 18S RNA were labelled with VIC. The Sham group was assigned the calibrator against which all other results were expressed as fold changes.

### 2.4. Western Blotting

Protein samples were separated on NuPage Novex 4%–12% Bis–Tris gels (Life Technologies, Carlsbad, CA, USA) and transferred to PVDF membranes (Pierce, Rockford, IL, USA). After blocking with 5% skim milk, the membranes were incubated with primary antibodies (microtubule associated 1A/1B light chain protein 3 (LC3A/B, 1:2000, Cat# 4108 S, Cell Signalling Technology, Danvers, MA, USA), manganese superoxide dismutase (MnSOD, 1:2000, Cat# 06-984, Millipore, MA, USA), glutathione peroxidase 1 (GPx1, 1:250, Cat# AF3798, R&D systems, Minneapolis, MN, USA), mitochondrial dynamin like GTPase (OPA-1, 1:2000, Cat# NB110-55290, Novus Biotechnology, Centennial, CO, USA), dynamin related protein 1 (DRP-1, Cat# NB110-55237, 1:2000, Novus Biotechnology, Centennial, CO, USA), fission 1 protein (Fis-1, 1:500, Cat# FL-152, Santa Cruz Biotechnology, Dallas, TX, USA), EGF-like module-containing mucin-like hormone receptor-like 1 (F4/80, 1:1000 Cat# sab5500103, Sigma-Aldrich, St. Louis MO, USA)) followed by the corresponding secondary antibody (Abcam, Cambridge, UK). Bands were detected with SuperSignal West Pico Chemiluminescent substrate (Thermo Fisher Scientific, CA, USA) and Fujifilm LAS-3000 (Fujifilm, Tokyo, Japan) and then quantified with ImageJ (National Institutes of Health, Bethesda, MD, USA). Results were expressed as a ratio against the housekeeping protein β-actin or cytochrome c oxidase subunit (COX) IV.

### 2.5. Immunohistochemistry

Formalin fixed, paraffin embedded livers were sectioned at 5 µm. Sections were deparaffinised and rehydrated in xylene and decreasing grades of ethanol. Antigen retrieval was performed [7] before the sections were incubated with anti-rabbit collagen III (1:50, Cat# NB600-594, Novus Biotechnology, Centennial, CO, USA) or cluster of differentiation 68 (CD68) (1:600, Cat# OABB00472, Aviva Systems Biology, San Diego, CA, USA) primary antibody and horseradish peroxidase anti-rabbit Envision system (Dako Cytochemistry, Tokyo, Japan). Sections were counterstained with haematoxylin and quantified with ImageJ (National Institutes of Health, Bethesda, MD, USA). 

### 2.6. Second Harmonic Generation

Images were acquired on a Leica SP8 multi-photon microscope equipped with a Mai Tai DeepSee laser tuned to 930 nm. Second harmonic generation signal was collected between 455–475 nm using a HyD detector in photon counting mode and the autofluorescence was collected from 480–600 nm. Channel separation and stitching were performed within the Leica Application Suite X software (Leica Microsystems, Wetzlar, Germany) controlling the microscope, before being deconvolved with Huygens Professional version 18.04 (Scientific Volume Imaging, Hilversum, The Netherlands). The data was 3D median filtered to remove noise, the background subtracted, and the amount of collagen quantified using ImageJ (National Institutes of Health, Bethesda, MD, USA). A Fast Fourier Transform (FFT) filter was applied to the autofluorescence channel to remove stitching artifacts. Image projections are summed slices of the 3D z stack, and to enable visualisation of the total amount of collagen, spanning the entire dynamic range in a single image, a gamma correction of 0.2 was applied.

### 2.7. Mitochondrial Density and ROS

Frozen livers were sectioned to quantify mitochondrial density and ROS levels. Liver mitochondria were visualised using MitoTracker Green (Thermo Fisher Scientific, Waltham, MA, USA) and images were acquired at 488 nm excitation wavelength and detected in the 510–550 nm emission range as previously reported [14]. For total reactive oxygen species (ROS), CellROX Deep Red (Thermo Fisher Scientific, Waltham, MA, USA) was used and images were acquired at 633 nm excitation wavelength and detected in the 640–680 nm emission range. All imaging parameters including laser intensities, photomultiplier tubes voltage and pinholes were kept constant during imaging. The MitoTracker and CellRox images were overlayed to provide information on mitochondrial specific ROS. Data were generated using ImageJ (National Institutes of Health, Bethesda, MD, USA).

### 2.8. Lipidomics

Lipids were extracted from frozen liver tissue using methyl-tert-butyl ether [20]. Cryohomogenised liver tissues were incubated with methanol and then methyl-tert-butyl ether, before centrifugation to induce phase separation. The top organic phase was dried and then resuspended in methanol:isopropanol (2:1) mixture.

Using a Vanquish Ultra Performance Liquid Chromatography (UPLC) system (Thermo Fisher Scientific, Waltham, MA, USA), 2 µL of the sample was loaded at 400 mL/min onto an Accucore™ C30 (2.1 × 150 mm, 2.6 μm) column with a 70:30 A:B solvent mix (A: 60% Acetonitrile (ACN)/40% H_2_O containing 10 mM ammonium formate and 0.1% formic acid (FA). B: 90% isopropyl alcohol (IPA)/10% ACN containing 10 mM ammonium formate and 0.1% FA). Retained lipids were eluted from the column and into the Q Exactive Plus mass spectrometer (Thermo Fisher Scientific, Waltham, MA, USA) using the following program: 30–43% B over 2 min, 43–55% B over 0.1 min, 55–65% B for 9.9 min, 65–85% for 6 min, 85–100% B over 2 min, held at 100% B for 5 min, 30%–100% B over 0.1 min and re-equilibration for 5 min. The eluting peptides were ionised. A data dependent MS/MS and selected ion monitoring (dd-MS2 – dd-SIM) experiment was performed, with a survey scan of 250–1200 Da performed at 140,000 resolution with an AGC target of 1 × 10^6^ and maximum injection time of 100 ms. Ions listed on the inclusion list were selected for fragmentation in the higher-energy collisional dissociation cell using an isolation window of 1.0 m/z, an AGC target of 1 × 10^5^ and maximum injection time of 100 ms. Fragments were scanned in the Orbitrap analyser at 17,500 resolution and the production fragment masses measured over a mass range of 200–2000 Da. The mass of the precursor peptide was then excluded. The data files were searched using the Lipidex software package [21] in conjunction with Compound Discoverer (Thermo Fisher Scientific, Waltham, MA, USA).

### 2.9. Statistical Analysis

Results are expressed as mean ± SEM and were analysed using one-way ANOVA with Fisher’s least significant post hoc test if the data were normally distributed. If the data were not normally distributed, they were log transformed to achieve normality of distribution before analysis (GraphPad Prism 7.03, San Diego, CA, USA). *p* < 0.05 was considered the threshold for statistical significance.

## 3. Results

### 3.1. Body and Liver Weights

Birth weight and body weight can be used as an indication of intrauterine and postnatal development in mice. Male offspring from SE mothers had lower birth weight compared to male offspring from Sham mothers, which was normalised when MitoQ was administered to the SE mothers (*p* < 0.01, SE + MQ vs. SE, Table 1). At 13 weeks, SE offspring remained smaller compared to the Sham offspring (*p* < 0.05), which was reversed in the SE + MQ group (*p* < 0.01, Table 1). SE offspring also had reduced liver weights when compared to the Sham offspring (*p* < 0.05) at 13 weeks; again, this was reversed by maternal MitoQ supplementation (*p* < 0.01, SE + MQ vs. SE, Table 1). However, when liver weights were expressed as a percentage of body weight, there were no differences among the three groups. Maternal SE resulted in intrauterine growth restriction in male offspring, which was reversed by MitoQ supplementation during pregnancy.

### 3.2. Glucose Metabolic Markers

**Figure 2:**
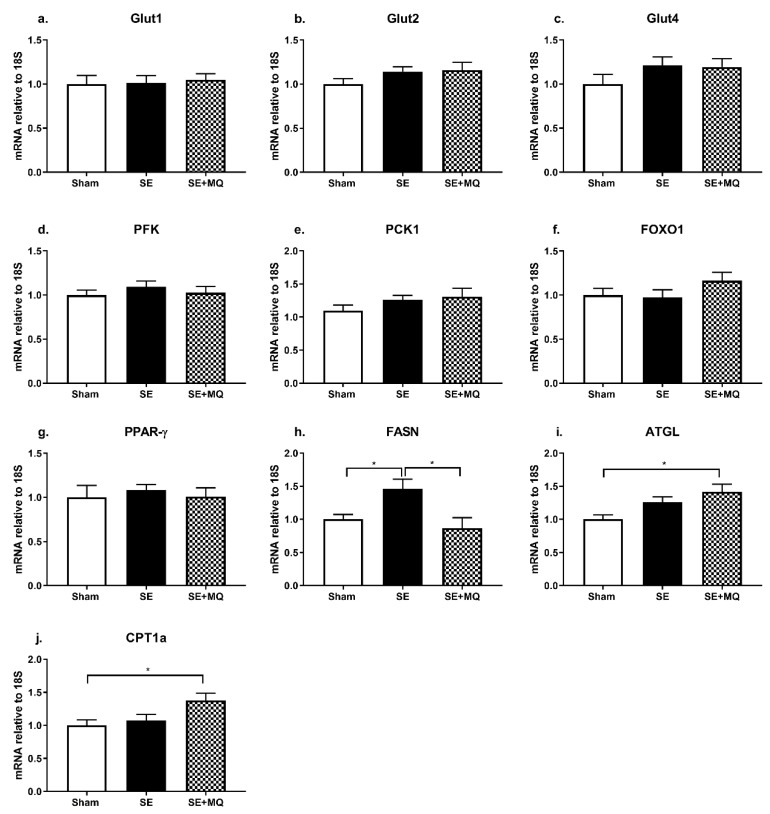
Liver mRNA expression of markers involved in glucose metabolism (**a–g**) and lipid metabolism (**h–j**) in 13 weeks old male offspring. Results are expressed as mean ± SEM (*n* = 6–8). * *p* < 0.05. ATGL: adipose triglyceride lipase, CPT1a: carnitine palmitoyltransferase 1a, FASN: fatty acid synthase, FOXO1: forkhead box protein O1, Glut: glucose transporter, PFK: phosphofructokinase, PPAR-γ: peroxisome proliferator-activated receptor gamma, SE: cigarette smoke exposure, SE + MQ: SE with MitoQ supplementation.

Impaired glucose clearance is associated with insulin resistance and type 2 diabetes. The IPGTT demonstrated that blood glucose was increased in the SE group compared to Sham group (*p* < 0.05) which was normalised in the SE + MQ group (*p* < 0.05 vs. SE, Table 1). However, hepatic expression of glucose transporters (Glut1, Glut2, and Glut4), glycolysis marker phosphofructokinase (PFK), gluconeogenesis markers (phosphoenolpyruvate carboxykinase 1 (PCK1), and forkhead box protein O1 (FOXO1)), and liver insulin sensing marker peroxisome proliferator-activated receptor gamma (PPAR-γ) were not different among the groups (Figure 2a–g). Maternal MitoQ supplementation reversed glucose intolerance induced by intrauterine tobacco smoke exposure, however, hepatic glucose metabolic markers were not affected. 

### 3.3. Lipid Metabolic Markers

Since blood glucose was increased in the SE group and hepatic steatosis is closely associated with the development of type 2 diabetes, liver lipid content was evaluated. Hepatic triglyceride (TG) concentration was increased in the SE group compared to the Sham group (*p* < 0.05), which was normalised in the SE + MQ group (*p* < 0.05 vs. SE, Table 1). Hepatic fatty acid synthase (FASN) mRNA expression was significantly increased in the SE group compared to the Sham group (*p* < 0.05) which again was normalised in the SE + MQ offspring (*p* < 0.01 vs. SE, Figure 2h). Adipose triglyceride lipase (ATGL) and carnitine palmitoyltransferase 1a (CPT1a) mRNA levels were only increased in the SE + MQ group (*p* < 0.05 vs. Sham, Figure 2i,j). 

From lipidomics analysis, liver concentrations of certain phosphatidylethanolamine (PE) species, specifically PE 34:1, PE 38:1, PE 38:2, and PE 40:5 were increased in the SE group compared to the Sham group (*p* < 0.05), with PE 38:2 being normalised in the SE+ MQ group (*p* < 0.05 vs. SE, Table 1). No differences in other lipid species detected during lipidomics analysis were observed (see Table S2). In addition, plasma TG concentrations were not different among the three groups (Table 1). Overall, increased hepatic de novo lipogenesis could be responsible for elevated hepatic TG concentrations in the SE group. Furthermore, maternal MitoQ supplementation normalised liver TG concentration in the offspring via a normalisation of de novo lipogenesis and an increase in mitochondrial fatty acid β-oxidation. 

### 3.4. Liver Injury Markers

**Figure 3:**
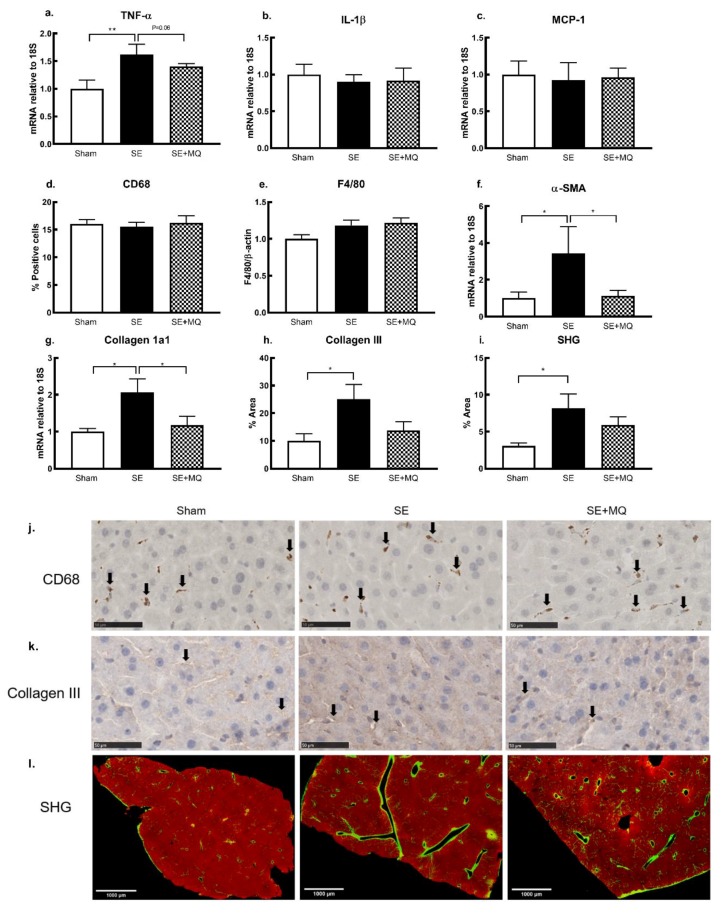
Markers of liver inflammation TNF-α, IL-1β (**a,b**), macrophage markers MCP-1, CD68 and F4/80 (**c–e**), stellate cell activation marker α- SMA (**f**) and fibrous deposition of collagen 1a1, collagen III, and total collagen (**g–i**) in 13 weeks old male offspring. Results are expressed as mean ± SEM (*n* = 4–8). * *p* < 0.05, ** *p* < 0.01. Representative images of hepatic CD68 (**j**, positive staining indicated by arrows) and collagen III (**k**, positive staining indicated by arrows) immunohistochemistry staining in 13 weeks old male offspring, bar = 50 µm. (**l**) Representative images of SHG showing total collagen staining after gamma correction (collagen is green/yellow), bar = 1000 µm. α-SMA: alpha-smooth muscle actin, CD68: cluster of differentiation 68, F4/80: EGF-like module-containing mucin-like hormone receptor-like 1, IL-1β: interleukin 1 beta, MCP-1: monocyte chemoattractant protein 1, SE: cigarette smoke exposure, SE + MQ: SE with MitoQ supplementation, SHG: second harmonic generation, TNF-α: tumour necrosis factor-α.

Plasma ALT activity and hepatic markers of inflammation and fibrosis are often elevated during liver injury. Plasma ALT was significantly increased in the SE group compared to the Sham group (*p* < 0.05); this was reversed in the SE + MQ group (*p* < 0.05 vs. SE, Table 1). Hepatic tumour necrosis factor (TNF)-α mRNA expression was significantly increased in the SE group compared to the Sham group (*p* < 0.01, Figure 3a) and was nearly normalised in the SE + MQ offspring (*p* = 0.06 vs. SE, Figure 3a). However, mRNA expression of interleukin (IL)-1β was not different between the groups (Figure 3b). Protein expression of surface macrophage markers, CD68 and F4/80, and mRNA expression of the macrophage chemokine, monocyte chemoattractant protein 1 (MCP-1), were not different among the three groups (Figure 3c–e). Stellate cell activity marker, α-smooth muscle actin (α-SMA), was increased in the SE group (*p* < 0.05 vs. Sham, Figure 3f) which was reversed in the SE + MQ group (*p* < 0.05 vs. SE, Figure 3f). The deposition of hepatic collagen 1a1, collagen III and total collagen was increased in the SE group compared to the Sham group (*p* < 0.05, Figure 3g–i), with collagen1a1 deposition being normalised in the SE + MQ group (*p* < 0.05 vs. SE, Figure 3g). Elevated liver injury markers in the offspring from the SE dams were mostly reversed by maternal MitoQ supplementation during pregnancy. However, this did not seem to be associated with changes in resident macrophages. 

### 3.5. Oxidative Stress and Mitochondrial Integrity

**Figure 4:**
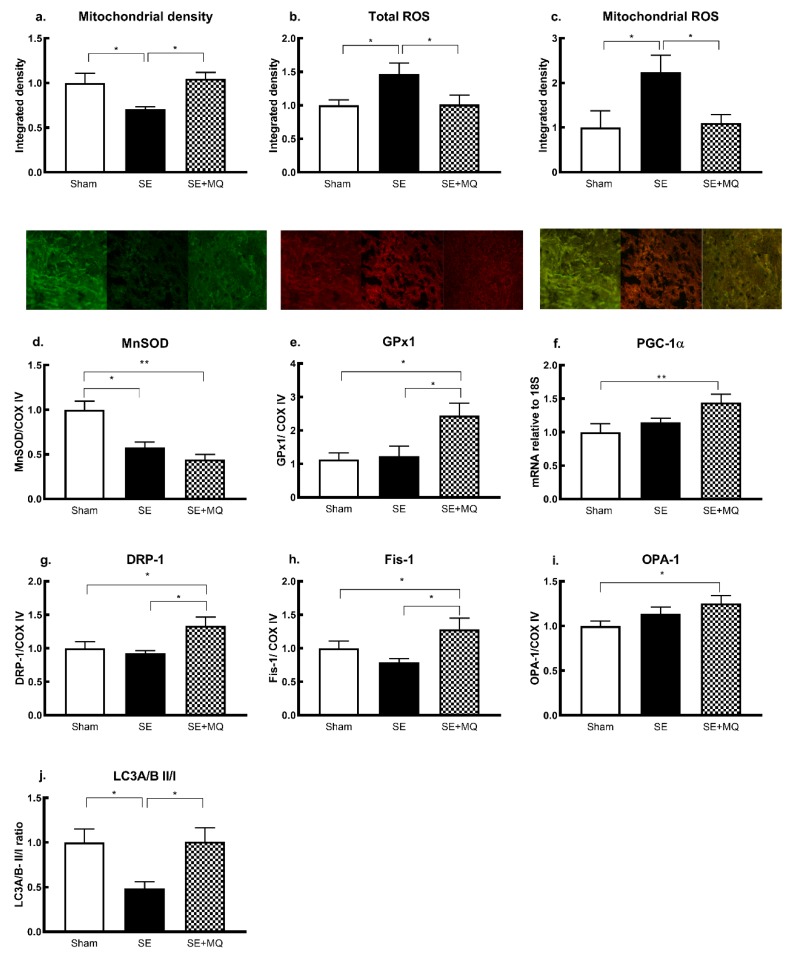
Hepatic mitochondrial density (**a**, green signal), ROS (**b**, red signal) and mitochondrial specific ROS (**c**, orange signal), endogenous mitochondrial antioxidants MnSOD (**d**) and GPx1 (**e**), marker of mitochondrial biogenesis PGC-1α (**f**) and markers of mitophagy (**g–j**) in 13 weeks old male offspring. Results are expressed as mean ± SEM (*n* = 6–8). * *p* < 0.5, ** *p* < 0.01. DRP-1: dynamin related protein 1, Fis-1: fission 1 protein, GPx1: glutathione peroxidase 1, LC3A/B II/I: microtubule associated 1A/1B light chain protein 3, MnSOD: manganese superoxide dismutase, OPA-1: mitochondrial dynamin like GTPase, PGC-1α: peroxisome proliferator-activated receptor gamma coactivator 1-α, ROS: reactive oxygen species, SE: cigarette smoke exposure, SE + MQ: SE with MitoQ supplementation.

Oxidative stress and mitochondrial damage are implicated in the development of metabolic disorders and hepatic steatosis. There was a significant decrease in mitochondrial density in the SE group compared to the Sham group (*p* < 0.05) which was normalised in the SE + MQ group (*p* < 0.05 vs. SE, Figure 4a). Furthermore, an increase in total ROS and mitochondrial ROS levels was observed in the SE group compared to the Sham group (*p* < 0.05), which was normalised in the SE + MQ group (*p* < 0.05 vs. SE, Figure 4b,c). Protein expression of the endogenous antioxidant MnSOD was reduced in both SE and SE + MQ groups compared to the Sham group (*p* < 0.05 and *p* < 0.01 respectively, Figure 4d); whereas, GPx1 protein was increased in the SE + MQ offspring compared to both the Sham and SE offspring (*p* < 0.05, Figure 4e). Mitochondrial biogenesis marker peroxisome proliferator-activated receptor gamma coactivator (PGC)-1a mRNA expression was increased in the SE + MQ group (*p* < 0.01 vs. Sham, Figure 4f). 

Both mitochondrial fission markers DRP-1 and Fis-1 were not significantly changed in the SE offspring, but increased in the SE + MQ group compared to both the Sham and SE groups (*p* < 0.05, Figure 4g,h). Mitochondrial fusion marker OPA-1 was not altered in the SE offspring, but significantly increased in the SE + MQ group (*p* < 0.05 vs. Sham, Figure 4i). The ratio of LC3A/B II to LC3A/B I was reduced in the SE group (*p* < 0.05 vs. Sham) but normalised in the SE + MQ group (*p* < 0.05 vs. SE, Figure 4j). Increased liver steatosis and damage in the SE offspring could be due to increased mitochondrial injury and impaired mitochondrial antioxidant defense capacity, leading to mitochondrial ROS accumulation. Increased liver markers of mitophagy and mitochondrial biogenesis by maternal MitoQ supplementation suggest an improvement in mitochondrial repair machinery. This was associated with a normalisation of mitochondrial ROS level, and improved hepatic steatosis and liver damage.

## 4. Discussion

The mechanisms underlying intrauterine underdevelopment and disease induced by maternal smoking are not well understood. Evidence from genetically modified mice suggests that these changes could be due to increased oxidative stress, rather than nicotine [22]. In this study, increased mitochondrial oxidative stress and resulting mitochondrial insufficiency are proposed as the underlying mechanisms. In our model, the offspring from the SE mothers had low birth weight, glucose intolerance, hepatic steatosis, inflammation, and fibrosis in adulthood. Maternal supplementation with the mitochondrial-targeted antioxidant, MitoQ, during gestation and lactation rescued body weight at birth, and decreased mitochondrial oxidative stress in the offspring’s liver, preventing hepatic changes in the offspring. As MitoQ reduces mitochondrial oxidative stress, our results suggest that it could be used as a novel treatment to prevent the metabolic dysfunction and liver damage induced by maternal SE.

In humans, intrauterine exposure to tobacco cigarette smoke leads to intrauterine growth restriction [23], insulin resistance [24], and hepatic steatosis [25], which were all observed in this study. In conjunction with our previous studies, this indicates the reproducibility and reliability of our model in reflecting human pathophysiology [1,8,9,15,25,26,27,28,29]. Cigarette smoke exposure causes vasoconstriction, limiting placental nutrient delivery and increasing the risk of low birth weight, leading to the development of type 2 diabetes and non-alcoholic fatty liver disease (NAFLD) in adulthood [25,26]. Inflammation is also important, as it leads to insulin resistance, interrupting lipid metabolism and causing liver steatosis and cellular damage, leading to fibrosis [30]. However, previous studies have only found modifications of genes related to fibrosis in the human male foetus, suggesting an increased risk of cirrhosis, but not steatosis [31]. In the present study, although hepatic glucose metabolic markers were not affected by maternal SE, de novo lipogenesis as indicated by upregulated FASN expression in adult SE offspring, contributing to increased liver triglyceride accumulation. Plasma PE levels are increased in patients with non-alcoholic steatohepatitis (NASH) [32]. We found increased PE levels in the SE offspring, accompanied by increased inflammation and fibrosis, suggesting an increased risk of NASH.

Of particular interest, maternal MitoQ supplementation reversed intrauterine growth restriction in the offspring. MitoQ has been shown to improve vascular function in both human and animal studies [33,34]. Therefore, we postulate that MitoQ may prevent the vasoconstriction induced by maternal SE, restoring nutrient supply to the foetus. This was reflected by an increase in birth weight in the SE + MQ offspring compared to the SE offspring, indicating better nutrient supply and intrauterine development. This may be due to increased uterine vasodilation since oxidative stress is closely related to smoking-induced vasoconstriction [35]. Previous studies have shown that MitoQ supplementation or increased cellular CoQ10 levels can reverse hepatic steatosis by inhibiting de novo lipogenesis and enhancing fatty acid β-oxidation [36,37,38]. Consistently, maternal MitoQ supplementation not only normalised systemic glucose metabolism and a marker of de novo lipogenesis (FASN), but also increased expression of lipid catabolism markers (ATGL and CPT-1α) in the offspring. 

Mitochondrial oxidative stress and injury have been observed in patients with NAFLD and NASH [39,40]. In this study, we identified mitochondrial oxidative stress and mitochondrial insufficiency as a critical mechanism in the foetal origin of hepatic steatosis and fibrosis resulting from maternal smoking. In addition, a similar situation was found in the brain and kidneys, associated with functional disorders [8,9,13,28]. We found maternal SE impaired markers of mitophagy, which may be due to both oxidative stress and lipid overaccumulation [41]. Mitochondrial oxidative stress is a well-known trigger of inflammation and in this study was reflected by increased production of TNF-α in the SE offspring. Interestingly, this did not appear to be the result of increased macrophage numbers (CD68 and F4/80 positive cells). Thus, the increase in TNF-α expression could be due to increased macrophage activity, or increased inflammatory responses within hepatocytes or stellate cells [42]. Reduced levels of the endogenous mitochondrial antioxidant MnSOD has been consistently observed in multiple organs from in utero cigarette smoke exposure in murine [13,14,43] and non-human primate models [44]. This study, together with those from a series of studies in the same model [13,14], have established the key role of systemic mitochondrial insufficiency and mitochondrial specific oxidative stress in foetal underdevelopment induced by maternal smoking [27]. 

Unlike other conventional antioxidants, MitoQ rapidly accumulates within the inner mitochondrial membrane to suppress oxidative stress [10]. In the MitoQ treated mothers, the offspring had a normal hepatic mitochondrial function, accompanied by reduced inflammation, fibrosis and lipid profiles. We suggest that this is an effect not driven by the inhibition of inflammation, as previous studies have found that depletion of Kupffer cells (resident liver macrophages) failed to protect the liver from fibrotic injury [33]; thus, normalising mitochondrial oxidative stress is the key. Oxidative stress may promote the activation of fibrogenic myofibroblasts, as indicated by α-SMA expression which was associated with ROS and fibrosis levels [45]. Here, there was an increase in the endogenous mitochondrial antioxidant GPx1, the mitochondrial biogenesis mediator PCG1a, and the mitochondrial self-renew machinery—DRP-1, Fis-1, OPA-1, and LC3A/B. However, another mitochondrial antioxidant MnSOD was not changed. It has been suggested that MitoQ mimics the function of MnSOD, which neutralises superoxide radicals. Therefore, there may be no need to increase MnSOD in the presence of MitoQ. However, the level of GPx1, which acts downstream of MnSOD, was increased, suggesting enhanced ROS scavenging capacity. Increased DRP-1 and Fis-1 by maternal MitoQ supplementation can facilitate the separation of healthy and damaged mitochondrial fragments due to oxidative injury. Increased OPA-1 can improve the recycling of healthy mitochondrial fragments to generate new mitochondria. The damaged fragments are further degraded by autophagosomes formed by LC3A/B to protect cells from their toxic effects [4]. Thus, taken together, maternal MitoQ supplementation restored mitochondrial integrity and oxidative stress level in the offspring’s liver and prevented the development of metabolic disorders. MitoQ also appears to protect offspring from liver steatosis better than the non-mitochondrial targeted antioxidant L-carnitine as we showed previously [27].

In this study, a low dosage of tobacco cigarette smoke was administered to the mothers, which may only apply to light smokers who continue to smoke during pregnancy. The effect of maternal MitoQ supplementation in mice exposed to high doses of cigarette smoke requires further investigation. In addition, the offspring were fed a balanced diet after weaning. Future studies can add a secondary insult (e.g., high fat diet or high carbohydrate diet [46]) to determine whether the risk of metabolic disorders in the liver can be mitigated.

We suggest that MitoQ can inhibit some of the detrimental effects of in utero cigarette smoke exposure on liver health. We are not suggesting that it is safe to smoke and use MitoQ, but in situations where mothers are unable to quit smoking there might be a beneficial effect of using MitoQ. Similarly, there might be efficacy against the detrimental effects of other endogenous inhaled oxidants, such as pollution or workplace noxious gasses. Although sold as an over-the-counter supplement, the safety of MitoQ during pregnancy has not been evaluated in humans. Nevertheless, the current study provides evidence for MitoQ supplementation in women who are unable to quit smoking during pregnancy to protect offspring from metabolic dysfunction.

## Figures and Tables

**Table 1:** Birth weight and parameters in 13 weeks old male offspring.

	Sham (*n* = 19)	SE (*n* = 20)	SE + MQ (*n* = 14)
Birth weight (g)	1.51 ± 0.03	1.30 ± 0.06 **	1.65 ± 0.05 ^##^
Body weight at 13 weeks (g)	25.2 ± 0.2	24.2 ± 0.2 **	25.1 ± 0.2 ^##^
Liver weight (g)	1.12 ± 0.019	1.05 ± 0.019 *	1.15 ± 0.02 ^##^
Liver weight (% of body weight)	4.42 ± 0.073	4.37 ± 0.079	4.55 ± 0.08
Liver triglycerides (mg/g liver)	4.08 ± 0.41	6.03 ± 0.49 *	4.04 ± 0.61 ^#^
Liver PE 34:1 intensity (cps)	121,000 ± 8900	203,000 ± 29,000 *	170,000 ± 19,000
Liver PE 38:1 intensity (cps)	13,100 ± 1600	21,700 ± 3000 *	17,000 ± 1900
Liver PE 38:2 intensity (cps)	12,500 ± 2400	23,000 ± 4700 *	12,700 ± 1200 ^#^
Liver PE 40:5 intensity (cps)	88,200 ± 7000	156,000 ± 25,000 *	104,000 ± 15,000
Plasma triglycerides (mg/mL)	2.36 ± 0.19	2.31 ± 0.21	2.40 ± 0.29
Plasma ALT (U/L)	8.03 ± 1.1	11.2 ± 0.9 *	7.75 ± 0.7 ^#^
Plasma insulin (ng/mL)	0.0702 ± 0.014	0.103 ± 0.083	0.111 ± 0.010
AUC of IPGTT (mM·min)	1150 ± 20	1280 ± 41 *	1130 ± 38 ^#^

Data are expressed as mean ± SEM (*n* = 14–20 for birth weight, body weight at 13 weeks, liver weight, and AUC of IPGTT; *n* = 6–8 for liver triglycerides, plasma triglycerides, plasma ALT, plasma insulin; *n* = 5 for liver PE). * *p* < 0.05, ** *p* < 0.01 vs. Sham. ^#^ *p* < 0.05, ^##^ *p* < 0.01 vs. SE. ALT: alanine aminotransferase, AUC: area under the curve, IPGTT: intraperitoneal glucose tolerance test, PE: phosphatidylethanolamine, SE: cigarette smoke exposure, SE + MQ: SE with MitoQ supplementation.

## Supplementary Materials

The following are available online at https://www.mdpi.com/2072-6643/11/7/1669/s1, Table S1: TaqMan Probe sequence (Life Technologies, CA, USA) used for rt-PCR, Table S2: Lipid species in the liver detected by lipidomics analysis.
