# Supplementary material for: A Mitochondrial Specific Antioxidant Reverses Metabolic Dysfunction and Fatty Liver Induced by Maternal Cigarette Smoke in Mice
Source: Nutrients. 2019 Jul 21;11(7):1669. doi: 10.3390/nu11071669 (PMC6682890; doi:10.3390/nu11071669)
Supplement: Supplementary file 1 [file nutrients-11-01669-s001.pdf]

Table S1. TaqMan Probe sequence (Life Technologies, CA, USA) used for rt-PCR.

Gene	NCBI References	Probe Sequence	ID
ACTA2	NM_007392.3	TAGCCCTGGCCTAGCAACACTGATT	Mm00725412_s1
ATGL	NM_025802.3	CCAAGACTGAATGGCTGGATGGCAA	Mm00503040_m1
COL1A1	NM_007742.3	AGTCTACATGTCTAGGGTCTAGACA	Mm00801666_g1
CPT1a	NM_013495.2	TTCCAGGAGAATGCCAGGAGGTCAT	Mm01231183_m1
FASN	NM_007988.3	AGCAATTGTGGATGGAGGTATCAAC	Mm00662319_m1
FOXO1	NM_019739.3	TCGGCGGGCTGGAAGAATTCAATTC	Mm00490671_m1
IL1B	NM_008361.3	TCCTTGTGCAAGTGTCTGAAGCAGC	Mm01336189_m1
PCK1	NM_011044.2	CCGTAGACCTGAAGGTGTCCCCCTT	Mm01247058_m1
PGC1a	NR_027710.1	CTGGAACTGCAGGCCTAACTCCTCC	Mm01208835_m1
PFK	NM_008826.4	GCGGTGATGCGCAAGGTATGAATGC	Mm00435587_m1
PPAR-γ	NM_0011273330.1	ATGCTGTTATGGGTGAAACTCTGG	Mm01184322_m1
SLC2A1	NM_011400.3	CATCGCCCTGGCCTTGCTGGAACGG	Mm00441480_m1
SLC2A2	NM_031197.2	CCGCCTCCCCCGGCGCGCACACACC	Mm00446229_m1
SLC2A4	NM_009204.2	TGGCTCTGCTGCTGCTGGAACGGGT	Mm00436615_m1
TNFα	NM_013693.2	CCCTCACACTCAGATCATCTTCTCA	Mm00443259_g1

Acta2: α-smooth muscle actin*, Atgl*: Adipose triglyceride lipase, *Cd68*: Cluster of differentiation 68, *Cpt1a*: Carnitine palmitoyltransferase 1a, *Fasn*: Fatty acid synthase, *Foxo1*: Forkhead box protein O1, *Pgc1a*: Peroxisome proliferator-activated receptor gamma coactivator 1-α, *Pck1*: Phosphoenolpyruvate carboxykinase 1, *Ppar-γ*: Peroxisome proliferator-activated receptor gamma, *Pfk*: Phosphofructokinase, SE: cigarette smoke exposure, SE+MQ: SE with MitoQ supplementation, *Slc2a1*: Glut1, *Slc2a2*: Glut2, *Slc2a4*: Glut4, *Tnfα*: Tumor necrosis factor-α.

Table S2. Lipid species in the liver detected by lipidomics analysis.

RT (min)	Identification	Sham Intensity (cps)	SE Intensity (cps)	SE+MQ Intensity (cps)
13.191	PE 36:5	4748±1345	5524±1085	5834±835
13.359	PE 38:7	93671±12543	161508±39763	91261±11121
13.847	PE 40:8	42933±8000	65586±16331	49017±5949
13.855	PE 36:5	50681±8323	95868±25379	48692±6984
13.855	PE 38:8	3921±617	7600±1921	4520±644
14.15	PE 34:3	21528±4301	33784±7186	21459±2996
14.192	PE 38:7	18933±3512	31311±7458	22078±3079
14.226	PE 37:6	12163±2852	16650±3625	15457±2265
14.495	PE 38:8	10385±1851	13684±2959	11249±1591
14.522	PE 39:7	32430±5655	49481±10664	44258±5233
14.53	PE 40:7	3727±339	5966±1318	4811±819
14.604	PE 36:4	63487±14994	85918±18161	67706±7782
14.629	PE 38:6	178755±35163	300132±74613	184502±21694
14.705	PE 34:3	23789±9463	22096±2078	29859±3641
15.041	PE 34:3	4766±1050	6287±1533	5190±665
15.148	PE-NMe2 38:6	57240±14340	61935±14019	53637±8398
15.215	PE 35:3	2159±563	2898±597	2288±221
15.369	PE 38:6	4694148±713042	6099049±982543	5619157±736726
15.377	PE 40:9	227368±27913	169240±24766	202810±36130
15.503	PE 42:10	64210±9180	55613±8582	62573±11614
15.522	PE 40:7	1333445±295460	1899879±341033	1498967±183253
15.654	PE 32:1	13533±2019	26077±5536	14686±2265
15.915	PE-NMe2 34:2	5878±1238	10478±2709	6670±806
15.996	PE 40:8	84216±14709	98276±14505	83927±10770
16.017	PE 39:6	9570±1516	17099±3625	14245±1853
16.051	PS 40:6	782327±174683	693019±160100	683561±102890
16.124	PE 34:2	734740±152746	855722±170424	762476±84106
16.287	PE 36:3	444326±99436	589139±128140	469697±49028
16.302	PE 38:6	24721±4917	25200±3944	22791±2676
16.445	PE 39:6	58586±14526	69779±14877	68037±10619
16.663	PE 33:1	1700±216	2843±510	2521±325

16.665	PE 36:3	54091±9900	86536±17012	65324±7676
16.705	PE 40:8	12774±2625	14941±2787	14120±2118
16.814	PE 40:6	14647±2268	19705±3870	14784±1709
16.827	PE 40:7	2962±432	5226±1147	3564±334
16.828	PE 38:4	39291±7762	63841±18540	35658±4058
16.91	PE 39:7	4990±766	5080±634	5437±897
16.981	PS 36:2	15169±6297	13674±3983	9675±2478
17.036	PI 38:3	60653±13052	66282±14537	55600±5942
17.19	PE 35:2	24727±5411	20516±3955	23755±3986
17.292	PE 36:3	5024±942	6784±1564	4945±553
17.439	PA 34:1	9671±2688	9476±2536	5726±1157
17.486	PE 40:6	2225961±388150	2113916±383568	1962195±288930
17.493	PE 42:9	132729±18044	102633±14093	129702±25499
17.621	PE 34:1	121270±8910	203223±29207*	169945±18992
17.845	PE 40:7	292044±43221	240169±32748	261743±38716
17.886	PE-NMe2 36:2	5692±1199	5647±1077	5463±627
17.89	PE 40:5	88238±6974	156020±25363*	103893±15011
18.013	PE 36:2	460362±99622	610928±109655	573191±83595
18.182	PE 41:6	22791±4829	21557±3433	23453±4001
18.257	PE 35:1	6859±2220	6509±868	6961±1131
18.282	PE 40:5	23876±6341	27674±4359	24190±3495
18.287	PE 38:3	114568±22675	170374±24083	129407±16706
18.289	PE 40:6	16013±2832	19184±2866	17891±2417
18.522	PE 37:2	9584±2043	8466±1211	9572±1399
18.541	PE 40:4	17766±4039	23240±4281	19575±2725
18.668	PE 38:2	12464±2378	23058±4678*	12675±1193#
18.687	PE 36:1	64954±9634	93258±14868	82146±11525
18.891	PE 38:2	19272±1728	21991±2886	20370±1697
19.034	PE 40:3	2209±320	3227±645	2564±390
19.231	PE 38:1	13103±1581	21703±3003*	17024±1922
19.236	PE 40:2	2940±1066	3198±1338	3253±761
20.153	TG 18:3_18:3_18:3	15344±2221	43898±19091	39434±8783
20.361	TG 42:0	9675±1220	6140±1829	8758±1481
20.372	TG 14:0_16:0_18:3	169454±46336	374367±140958	841122±549469
20.437	TG 16:0_18:3_18:2	1387906±307583	2552634±775373	3976208±1928056
20.711	TG 16:0_18:2_18:1	8074384±1610661	9438356±2154155	10861013±3147549
20.826	TG 54:3	4092231±481959	7124186±2264960	8524145±2820249
20.835	TG 52:2	7229942±1500714	7174353±848949	7261535±1493622
20.856	TG 18:2_18:1_20:1	653822±69556	924291±212524	1232330±257067
20.967	TG 18:1_18:1_20:1	1031411±142735	1767167±556538	1984807±772490
21.17	TG 52:0	24053±4113	55860±19912	29831±11212
21.215	TG 18:1_18:1_21:0	21682±5595	40896±15747	39038±18587
21.297	TG 18:1_18:1_24:1	44775±10731	65446±20602	69983±29349

Data are expressed as mean ± SEM (n=3-5). *P<0.05 vs Sham. #P<0.05 vs SE. PA: phosphatidic acid, PE: phosphatidylethanolamine, PI: phosphatidylinositol, PS: phosphatidylserine, SE: cigarette smoke exposure, SE+MQ: SE with MitoQ supplementation, TG: triglyceride.
